# Understanding HCMV Latency Using Unbiased Proteomic Analyses

**DOI:** 10.3390/pathogens9070590

**Published:** 2020-07-20

**Authors:** Emma Poole, John Sinclair

**Affiliations:** Department of Medicine, University of Cambridge, box 157, Level 5 Addenbrooke’s Hospital, Hills Road, Cambridge CB2 0QQ, UK; js152@cam.ac.uk

**Keywords:** human cytomegalovirus, proteome, latency

## Abstract

Human cytomegalovirus (HCMV) establishes either a latent (non-productive) or lytic (productive) infection depending upon cell type, cytokine milieu and the differentiation status of the infected cell. Undifferentiated cells, such as precursor cells of the myeloid lineage, support a latent infection whereas terminally differentiated cells, such as monocytes or dendritic cells are an environment conducive to reactivation and support a lytic infection. The mechanisms which regulate HCMV in either a latent or lytic infection have been the focus of intense investigation with a view to developing novel treatments for HCMV-associated disease which can have a heavy clinical burden after reactivation or primary infection in, especially, the immune compromised. To this end, a number of studies have been carried out in an unbiased manner to address global changes occurring within the latently infected cell to address the molecular changes associated with HCMV latency. In this review, we will concentrate on the proteomic analyses which have been carried out in undifferentiated myeloid cells which either stably express specific viral latency associated genes in isolation or on cells which have been latently infected with virus.

## 1. Introduction

Human cytomegalovirus (HCMV) is a species-specific pathogen which is carried by approximately 60–80% of the population, depending on demographics [1]. Front-line anti-virals for HCMV are limited due to poor bioavailability and drug resistance. Additionally, currently available drugs target the lytic phase of infection rather than the latent phase (the phases of the lifecycle are described in detail below) [2,3]. Therefore, the need to target latently infected cells could be crucial, particularly in settings such as solid organ or stem cell transplantation where latent virus in the graft itself can be transferred to an immune-suppressed donor or a seropositive graft recipient’s own virus can reactivate. In this review, we will discuss how understanding the cellular changes which occur during latency, by studying the latency-induced proteome, may not only lead to an enhanced understanding of the mechanisms of viral latency and reactivation but also aid the development of novel therapeutics to target the latent virus reservoir. For instance, latently infected cells can be killed by targeting latency-associated changes in cellular proteins (such as virally induced changes in expression of the cellular MRP-1 protein, detailed below) or killed by targeting the viral protein expressed during latency directly (as detailed for US28, below). Other identified changes have also highlighted mechanisms used during latency to optimize conditions for the virus, such as regulation of cellular genes S100A8/A9 and HCLS1.

## 2. HCMV Lytic and Latent Lifecycle

HCMV is a large double-stranded DNA virus with a protracted lytic lifecycle; up to 72 h in some cell types. This commences with immediate early (IE) gene expression, then early (E) gene expression and finally, late gene expression (L) [4]. The lytic lifecycle can take place in a wide range of terminally differentiated cell types, including fibroblasts, macrophages’ endothelial cells, resulting in full productive virus replication. The ability of the virus to establish a lifelong infection in the host is, at least in part, due to the ability of the virus to also be able to establish a latent infection during which time viral genome is carried in cells but IE gene expression is suppressed and infectious virions are not produced [5]. One established site of HCMV latency is in the cells of the myeloid lineage [5] where, in vivo, virus is carried in myeloid progenitors such as CD34+ progenitor cells and their derivative CD14+ monocytes. Then, as the cells differentiate along the myeloid lineage to terminally differentiated cell types such as macrophages or dendritic cells, the virus reactivates leading to the initiation of IE gene expression, the full temporal lytic gene cascade and ultimately, progeny virions [5].

### 2.1. Viral Gene Expression during HCMV Latency

Whilst latent infection in myeloid progenitor cells is marked by general suppression of IE gene expression and a lack of infectious virion production, the exact latency-associated viral transcription programme is far from clear. A number of studies have attempted to define the viral genes which are expressed during latency. These analyses used targeted primers for known viral genes suspected to be expressed during latency to detect viral transcripts in latently infected cells. Such studies have led to the identification of viral genes such as UL138, US28, UL144, UL81-82ast (LUNA) and UL111A (viral IL10) [6,7,8,9,10,11]. More recently, unbiased screening has identified different subsets of viral genes in both experimentally and naturally latent myeloid cells [12,13,14,15,16]. HCMV also encodes a number of viral miRNAs [17] which have been shown not only to be expressed during lytic infection but also during latency [18,19,20]. Therefore, whilst the exact viral gene expression profile during latent infection has not been categorically defined, it is now clear that, far from the genome being carried silently in latently infected cells, latent infection is accompanied by the expression of a number of viral genes which are likely to affect cellular gene expression to optimise latent carriage of the virus.

### 2.2. Cellular Transcriptome Analysis of HCMV Latency

Changes in the cellular RNAs resulting from latent infection of myeloid progenitors have included analyses of changes in the cellular miRNAome [19,21,22] as well as changes in cellular mRNAs based on either bulk or single cell RNA analyses [23,24,25,26,27,28]. These studies have led to the identification of a number of ways in which the virus manipulates host cell RNA expression during latency. For example, the downregulation of cellular hsa-miR-92a leads to a manipulation of the chemokine CCL8 [29], a chemokine previously identified to be upregulated during latency in the latency associated secretome [30]. It also appears that the viral genes expressed during latency regulate cellular genes involved in immune suppression and can drive latently infected myeloid cells into an anergic state [25,28]. Additionally, virus binding to monocytes has been shown to alter their transcriptome, changing the phenotype of the cells by, for example, regulating PI3 kinase and Erk/ MAP kinase signaling [24]. Indeed, it has been suggested that the binding of HCMV to the cell membrane causes reprogramming of monocyte survival and differentiation [31]. Some transcriptome analyses during HCMV latency have identified changes of genes involved in cell survival and immune regulation such as PEA-15 [23], which has since been validated and a mechanism defined [32,33]. Changes in MHC class II [23] during latency have also been identified and at least one mechanism for this change has been defined [34,35]. These studies have been informative but one way to obtain a more comprehensive understanding of changes in protein expression during latency is to analyse virally induced changes in the total proteome in an unbiased manner.

## 3. Studying the Proteome

During lytic infection, temporal changes in the viral proteome [4] as well as changes in the cell proteome mediated by lytic infection have been extensively analysed [4,36]. Such studies have been expanded upon, using for instance shRNA or CRISP-R libraries, to identify mechanisms by which viral-viral or viral-cellular protein–protein interactions regulate both cellular and viral gene expression during lytic infection [4,37]. Such high-throughput approaches to understanding HCMV lytic infection have been recently reviewed [37,38,39]. In this review, we discuss total proteomic analyses that have been carried out to interrogate cellular changes during HCMV latency.

### 3.1. Total Proteomic Changes during HCMV Latency

One difficulty when analysing total proteomic changes during latency is that the levels of infection are often low and it can be difficult to identify the latently infected cell in a mixed population. One way to address this is to generate a virus which expresses a fluorescent tag during a latent infection to allow the latently infected cells to be separated by FACS from the total cell population. Routinely, GFP expression under control of the SV40 promoter has been employed by a number of groups to identify latently infected cells. However, GFP expression can tend to wane over time and so alternative fluorescent tags to mark latently infected cells have also been derived such as SV40-mCherry or GATA2-mCherry in which mCherry is under the control of the GATA2 transcription factor, which is known to activate a number of viral latency-associated genes in undifferentiated myeloid cells. Proteomic studies on such cells have led to the identification of a number of cellular processes which are affected by HCMV latent infection of myeloid cells [35,40,41].

### 3.2. HCLS1

One cellular protein which is substantially upregulated during HCMV latent infection of CD14+ monocytes is Haematopoietic lineage cell-specific protein 1 (HCLS1). HCLS1 is expressed in cells of the haematopoietic lineage, such as CD34+ and CD14+ monocytes [42], and is known to be a cortactin homologue that is involved in the stabilisation of actin filaments thereby aiding myeloid cell motility and adhesion to the endothelial cell layer [43]. HCLS1 has also been reported to enhance transendothelial cell migration of NK cells [44]. All of these properties could potentially aid virus latent carriage and dissemination and it was found that latently infected cells are, indeed, more motile than uninfected monocytes and are more able to adhere to, and transit, the endothelial cell layer [40].

### 3.3. S100A8/A9

In addition to the identification of upregulated cellular proteins, proteomic analysis of latently infected monocytes has also identified a number of downregulated cellular proteins. Two of these, which were equally downregulated, were S100A8 and S100A9 which often function together as a dimer. One known property of S100A8/A9 is the ability to chemoattract cells, such as neutrophils [45]. Studies on latently infected cells showed that neutrophils are capable of killing latently infected cells at very high E:T (effector to target cell) ratios. At physiological E:T ratios, however, neutrophils do not kill latently infected cells which appears to be, at least in part, due to the downregulation of S100A8/A9 [41].

## 4. Proteome Changes Induced by Expression of Latency-Associated Proteins in Isolation

Though unbiased proteomic screens are a powerful way to interrogate latency-associated changes in cellular proteins, some targets could possibly be missed, especially because viral latency-associated gene expression is known to be very low in the latently infected cells. One way to address this is to overexpress putative latency associated genes in isolation. Another advantage of this targeted approach is that a screen which identifies changes in total cellular proteins induced by latency will not, inherently, identify which viral gene product has caused these changes. Therefore, a number of studies have analysed changes in myeloid cells which over-express specific latency-associated proteins. To date, this has been carried out for two viral genes known to be expressed during latency by overexpressing them in THP1 myelomonocytic cells, a cell type known to support HCMV latency and reactivation [7,46,47].

### 4.1. US28

US28 is a viral latency-associated transcript which, like all known viral latency associated genes, is also expressed during lytic infection. US28 mRNA has been detected during both experimental and natural latency [7,8,9,48] and encodes a membrane associated G-protein coupled receptor (GPCR) with seven transmembrane helices which is involved in a number of signalling pathways. During latency, US28 is expressed at the protein level as evidenced by the observations that latently infected cells can be killed by a drug which targets the US28 protein [49] as well as the observation that US28 protein plays a role in myeloid differentiation during HCMV latency in humanised mice [50].

In all but one study [7,8,25,35,41,48,50,51], US28 has been shown to be essential for the establishment and maintenance of latency in the myeloid lineage. One reported mechanism by which US28 regulates latency involves activation of STAT3 in the iNOS and NO pathway [25]. US28 is also known to be able to initiate different signalling pathways in a cell-type-specific manner. For instance, expression of US28 in undifferentiated myelomonocytic THP1 cells results in changes in the cellular phosphotome which are quite different to those induced by US28 in PMA-differentiated THP1 cells. For example, ERK, CREB and MSK signalling is downregulated by US28 in undifferentiated THP1 cells but, in terminally differentiated cells, these pathways are stimulated by US28 and such changes have been shown to differentially regulate expression from the MIEP [48,52].

Changes in the total cell proteome resulting from US28 expression in undifferentiated THP1 cells have also been analysed [35]. Of particular interest was the downregulation of a number of PYHIN family proteins by US28 which included MNDA (myeloid cell nuclear differentiation antigen) and IFI16 (Interferon-Inducible Protein 16). PYHIN proteins are so-called because of their common HIN200 domains and many members of this family of proteins have been shown to play a role in innate immune evasion. For example, IFI16 has been shown to be important for dsDNA virus sensing in certain cell types [53,54,55], can modulate viral gene expression during lytic HCMV infection [56,57] and is known to affect infection of other herpesviruses such as HSV-1 [58]. Additionally, IFI16 has been shown to activate NFkB signalling [59,60,61] and, during HCMV latent infection, downregulation of IFI16 by US28 appears to suppress NFkB-mediated activation of the MIEP, thereby helping to maintain latency [35].

### 4.2. UL138

UL138 is a latency-associated transcript which is known to be expressed during both experimental and natural latency [6,14,16,62] as well as lytic infection. The presence of UL138 is required for the establishment of latency in a number of in vitro systems [63] and the regulation and functions of UL138 during latency has been the subject of much research [64,65,66,67,68]. To help address the role of UL138 during latency, the changes in the cell plasma membrane resulting from its overexpression has been analysed [69]. Major changes in the plasma membrane profile were identified as a result of expression of UL138 and one protein which was identified as being massively down-regulated by UL138 was the Multi-Drug Resistance Protein-1 (MRP-1). MRP-1 acts as a drug transporter protein and pumps vinca alkaloids and other toxins out of the cell. It is unclear at present how this benefits the virus during latency although the regions for UL138-mediated MRP-1 degradation have been identified [68]. A direct effect of such downregulation of MRP-1 is that latently infected cells are less able to transport, e.g., vincristine out of the cell. Consistent with this, studies showed that latently infected cells were more sensitive to vincristine, and vincristine targeted and killed latently infected CD34+ cells [69]. The possible therapeutic potential of this is a topic of ongoing research.

## 5. Concluding Remarks

**Figure 1:**
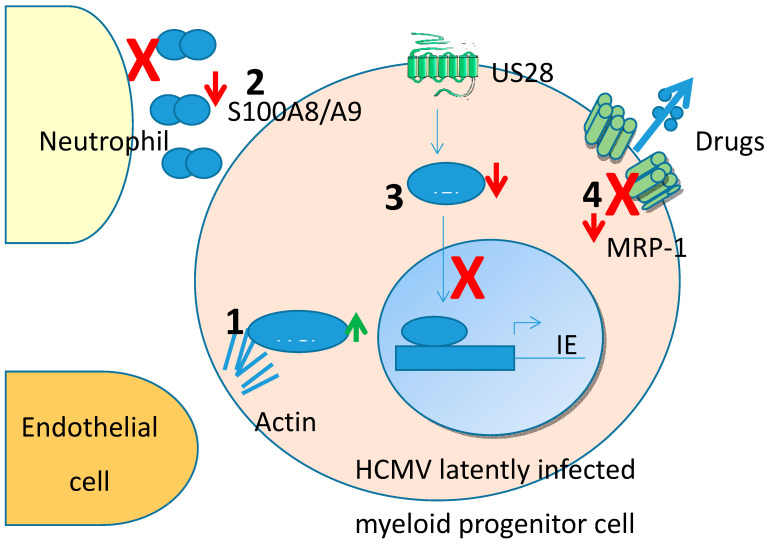
Cellular protein changes identified from unbiased proteomic screens in Human cytomegalovirus (HCMV) latency. (1) HCLS1 is upregulated which regulates actin stabilisation and, therefore, cell motility leading to enhanced endothelial cell attachment and transit. (2) S100A8/A9 are downregulated preventing the chemoattraction of neutrophils which could otherwise target and kill latently infected cells. (3) IFI16 is downregulated by latency-associated protein US28 and prevents NFkB activation of the MIEP and therefore helps aid the maintenance of latency. (4) Drug transporter MRP-1 is downregulated during latency by latency associated protein UL138 which renders cells sensitive to drug-mediated killing.

A comprehensive account of all changes in the cell resulting from HCMV latency is still some way off. However, such analyses have begun to give us some insight as to how the virus manipulates the cell during latency to optimise latent carriage. In this review, we have concentrated on changes to the cellular proteome. Many of these changes (summarised in Figure 1) have identified mechanisms by which latent infection modifies, transendothelial migration and avoidance of neutrophil killing. More targeted approaches have also identified changes in the cell brought about by expression of specific latency-associated viral genes in isolation and have shown how this results in changes in the cell that help evade host innate immune responses but other changes also render cells more sensitive to, e.g., potentially therapeutic toxins. Taken together, such analyses help us to understand the biological mechanism of latent virus carriage but also identify changes in the phenotype of latently infected cells that could become potential “Achilles heels” for them to be targeted therapeutically.
